# Collaborative optimization for train stop planning and train timetabling on high-speed railways based on passenger demand

**DOI:** 10.1371/journal.pone.0284747

**Published:** 2023-04-21

**Authors:** Yawei Li, Baoming Han, Peng Zhao, Ruixia Yang

**Affiliations:** 1 School of Traffic and Transportation, Beijing Jiaotong University, Beijing, China; 2 School of Automobile and Transportation, Tianjin University of Technology and Education, Tianjin, China; National Taiwan University of Science and Technology, TAIWAN

## Abstract

In recent years, with increasing passenger travel demand, high-speed railways have developed rapidly. The stop planning and timetabling problems are the core contents of high-speed railway transport planning and have important practical significance for improving efficiency of passenger travel and railway operation Dong et al. (2020). This study proposes a collaborative optimization approach that can be divided into two phases. In the first phase, a mixed-integer nonlinear programming model is constructed to obtain a stop plan by minimizing the total passenger travel time. The constraints of passenger origin-destination (OD) demand, train capacity, and stop frequency are considered in the first phase. In the second phase, the train timetable is optimized after the stop plan is obtained. A multiobjective mixed-integer linear optimization model is formulated by minimizing the total train travel time and the deviation between the expected and actual departure times from the origin station for all trains. Multiple types of trains and more refined headways are considered in the timetabling model. Finally, the approach is applied to China’s high-speed railway, and the GUROBI optimizer is used to solve the models in the above two stages. By analyzing the results, the total passenger travel time and train travel time decreased by 2.81% and 3.34% respectively. The proposed method generates a more efficient solution for the railway system.

## Introduction

High-speed railway has developed rapidly all over the world and won widespread support and popularity with the public for its many significant advantages, including punctuality, comfort, safety, and speed. At the same time, greater requirements are being placed on the transport plans of high-speed railways. Passenger demand grows at a steady rate and is often imbalanced, with extremely varied spatial mobility patterns and flow volumes. Moreover, passengers prefer to use direct services for modes with poor interchange conditions. Therefore, an efficient and scientific transport plan is essential to provide a high level of service that meets the needs of both passengers and operators.

**Fig 1:**
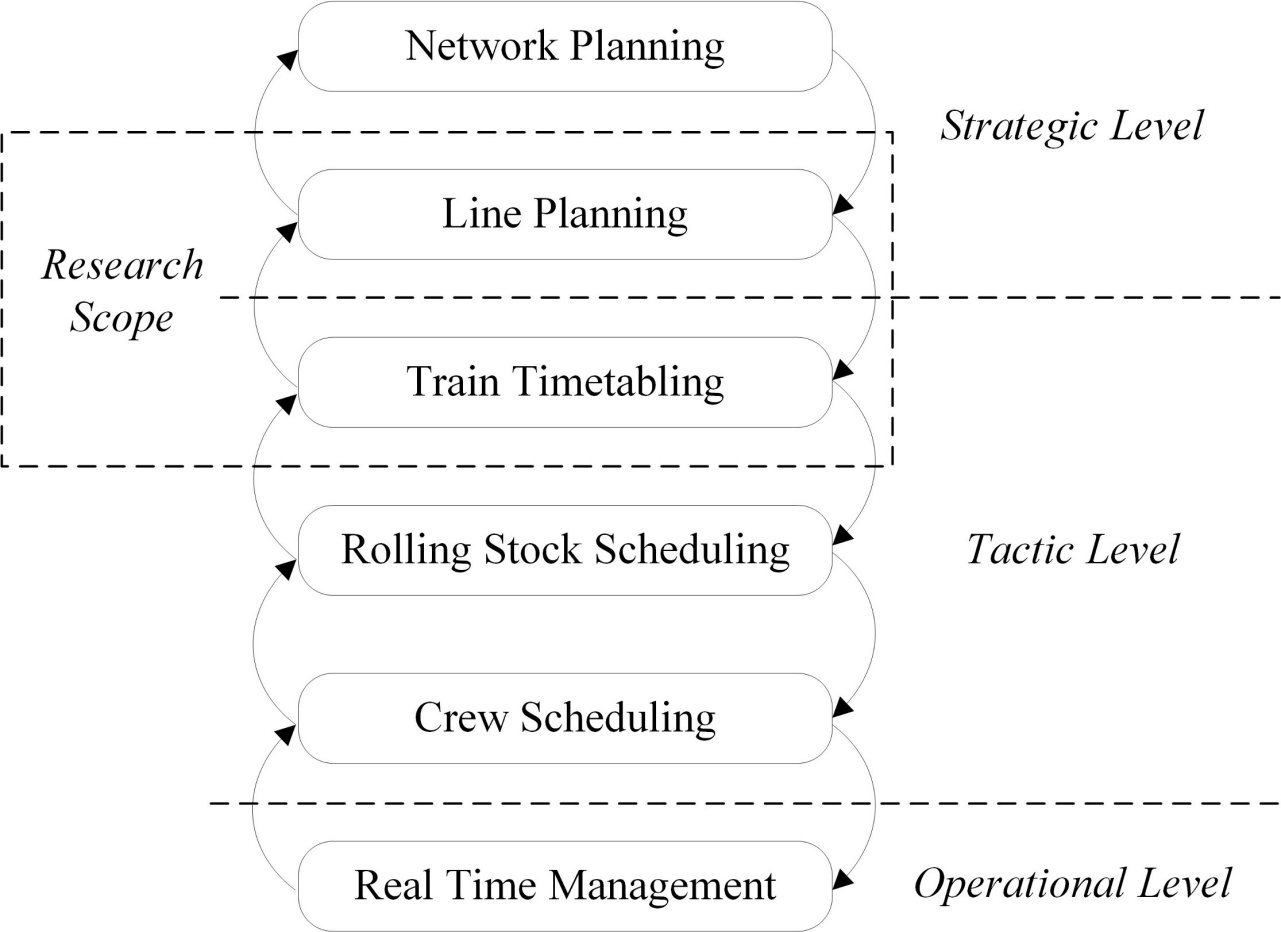
Railway transport planning process.

In general, the process of high-speed railway system train planning is divided into three levels, strategic, tactical, and operational, and at the same time, the process is decomposed into network planning, line planning, train timetabling, rolling stock scheduling, crew scheduling, and real-time management [1], as shown in Fig 1. As an important part of a transport plan, the train stop plan and timetable contain key elements such as the train stop pattern and train arrival, departure and pass times at stations. In this process, train operating schedules need to be adjusted when passenger demand changes. Once a prespecified stop plan is changed, a new timetable must be generated to accommodate the new train stop requirements. It is clear that this adjustment process can inherently complicate rail operations. Therefore, the design of effective methods to collaboratively optimize train stop plans and train timetables to better meet passenger demand is an issue that needs to be studied.

### Literature review

In recent years, scholars have performed a great deal of research on stop planning, timetabling and the integration of both. In this section, we review the models and methods of the three problems mentioned above.

#### Stop planning

Line planning requires specifying the number of trains, train types and their stop plans. Given the number of trains and operation parameters related to train types, it is worth studying to determine the stop plan for each train to fulfill passenger needs with the lowest possible cost. Bussieck et al. [2] introduced a mixed-integer linear programming formulation to maximize the number of direct travelers. A cutting plane approach was proposed to solve this problem. Claessens et al. [3] and Goossens et al. [4] solved the line planning problem by minimizing train operating costs. A further development called the multiline planning problem was proposed by Goossens et al. [5] later. They provided train lines with different halting patterns to solve the line planning problem. To minimize both passenger travel time and operation cost, Borndörfer et al. [6] developed a multi-objective optimization model and presented a column-generation algorithm to obtain its solution. Based on the classification of stations and trains, Fu et al. [7,8] proposed a two-stage train stopping optimization method which optimized higher-classification and lower-classification train stop plans respectively. Hu et al. [9] performed a circuity analyses of China’s high-speed rail network and found a series of reasonable shortest paths for trains. Parbo et al. [10] presented a bi-level model and a heuristic method to optimize stop patterns in the large-scale network. The lower level is a passenger assignment model, and the upper is the skip-stop optimization model. The results show that the proposed solution have a better performance in reducing passenger travel time. In summary, there are lots of studies to optimize stop plans with the given alternatives, and the specified or variable number of trains.

#### Timetabling

Generally, the obtained train stop plan is used as partial input to optimize the timetable, which specifies arrival and departure times of trains at all stations for passengers. Among relevant studies, timetables for railways can be divided into two categories: periodic and aperiodic timetables.

In view of a periodic timetable, Serafini and Ukovich [11] first proposed the periodic event scheduling problem (PESP). Some researchers focused on solving the PESP and then applying it to scheduling [12,13]. Kroon et al. [14] proposed a variable trip time model based on the PESP. Liebchen et al. [15,16] proposed an integer programming model based on graph theory to optimize the periodic timetable. The approach was applied to the Berlin metro to obtain a timetable with lower train operating costs and passenger waiting time. Many researchers have conducted studies on periodic timetabling considering robustness and stability [17,18]. Goverde et al. [19] used a max-plus model to perform real-time sensitivity and robustness analyses of large-scale periodic railway timetables. Yan et al. [20] proposed a railway timetable optimization model with overtaking and variable dwell and running times, aiming to improve the robustness. Sparing et al. [21] extended the PESP to a variable cycle time formulation and constructed a railway timetable optimization model to maximize railway network stability with flexible train orders, overtaking, and running times.

For aperiodic timetables, many integer programming models have been proposed and solved by various strategies, including the branch-and-bound method [22], Lagrangian relaxation [23] and other hybrid algorithms [24]. Caprara et al. [25,26] proposed a timetabling model based on graph theory and considered additional constraints in real-world applications, which is solved by a Lagrangian heuristic algorithm. Given section running time and dwell time, Niu et al. [27] developed a nonlinear optimization model containing binary variables indicating departure events and passenger loading to solve the timetabling problem in heavily congested urban rail corridors. However, the skip-stop strategy is not taken into account. To meet dynamic passenger demand, Barrena et al. [28,29] developed three linear optimization models to determine train departure times at stations and running speeds during sections. These aimed to minimize the average passenger waiting time. A fast adaptive large neighborhood search (ALNS) metaheuristic was introduced to solve large instances of the problem. D’Acierno et al. [30] studied the relationship between dwell times and passenger flow in the metro system. By providing a more accurate estimation of dwell times, the timetable could present a better robustness. Given dwell times and section running times, Niu et al. [31] developed a nonlinear optimization model to jointly synchronize train service times and effective passenger loading time windows at each station. They considered time-varying demand and found that train trajectories in the final schedule were closely synchronized with them. Robenek et al. [32] considered both periodic and aperiodic timetabling problem, with the aim of maintaining passenger satisfaction while maximizing the profit of train operators. The results show that the aperiodic timetable performs better for high density demand.

Integrated approach. In recent years, some scholars have performed research on integrated stop planning and timetabling approaches. To generate train stop plan and timetable simultaneously, Yang et al. [33] proposed a multiobjective integer linear programming model with the aim of minimizing the total dwell time and delay of train service times. To address the problem of optimizing high-speed railway stopping patterns and timetables, Yue et al. [34] constructed a linear programming model using the Lagrangian relaxation technology, intending to minimize train profit. A heuristic algorithm based on column generation was proposed to solve the problem in a real-world railway. An improved solution was obtained that increases the number of trains and profits. Yan et al. [35] optimized line plan and timetable by the two-stage approach. The first stage is used to obtain the optimal line plan, which is as the input for the second to optimize multi-period timetable with the aim of minimizing travel time, maximizing timetable robustness and minimizing number of overtakings. These two models works iteratively based on the designed feedback constraints. Considering the dynamic choice behavior of passengers, train service patterns and detailed timetables, infrastructure and rolling stock capacity, Meng et al. [36] constructed an integrated optimization model to determine train stops and timetable using a Lagrangian relaxation solution framework. Hao et al. [37] proposed an integer linear programming model for selecting an optimal minimum interval time, which takes symmetrical transport demand, train stopping schemes and train schedules into account. Dong et al. [38] constructed an integer nonlinear programming model to optimize train stop plan and timetable for commuter railway, without considering overtaking, which is solved by extended adaptive large-scale neighborhood search algorithm. The results showed that the proposed method can improve passenger travel efficiency and reduce train running time. Zhang et al. [39] considered different numbers of discrete time intervals as passengers’ expected departure time interval, and formulated an integer linear programming model to find the optimal stop plan and timetable with the aim of minimizing operation cost and train travel time.

Table 1 shows recent studies on stop planning and timetabling. According to the above literature review, a lot of research has been carried out on the stop plan, timetable and combination optimization. In summary, it is found that few research takes the relationship between train speed class and stop plan into account. For high-speed railways, there are often different types of trains in operation, which have a great impact on the travel of passengers. Although the headway constraint is included while timetabling, headways are not classified finely according the status of two consecutive trains. Variable dwell times and number of stop stations are considered in most studies while optimizing train timetable, but the time loss caused by acceleration and deceleration at the station is rarely taken into account. We also note that although some studies take minimizing the train delay as the objective. Each train is assumed to depart from the origin station not earlier than its expected departure time, which is too restrictive and inconsistent with the actual situation. Therefore, we need to find an effective and realistic way to combine train stop planning and train timetabling processes into a system-based optimization strategy where more complex situations and parameters need to be taken into account.

**Table 1:** Summary of relevant studies.

Study	Problem type	Model type	Objective	Train type	Headway type	Solution method
Fu et al. [8]	LP	MILP	Passenger travel time and unsatisfied passenger demand	Multiple	NA	Heuristics
Niu et al. [31]	TT	MINLP	Passenger waiting time	Single	Single	GAMS
Yang et al. [33]	SP & TT	MILP	Total dwelling time and total delay	Multiple	Single	CPLEX
Yue et al. [34]	SP & TT	MILP	Profit of train operation	Multiple	Single	Column-generation-based heuristic
Yan et al. [35]	LP & TT	MILP	Empty-seat-hours, passenger travel time, train travel time and timetable robustness	Single	Single	Iterative framework with GUROBI
Meng et al. [36]	TT	MILP	Transport profit	Single	Single	Lagrangian relaxation
Hao et al. [37]	SP & TT	MILP	Profit of train operation	Single	Single	Genetic algorithm
Dong et al. [38]	SP & TT	MINLP	Passenger travel efficiency and train running time	Single	Single	ALNS
Zhang et al. [39]	LP & TT	MILP	Operation cost and train travel time	Single	Single	GUROBI
This study	SP & TT	MINLP & MILP	Passenger travel time, train travel time and departure time deviation	Multiple	Multiple	GUROBI

**Problem type:** LP—line planning; SP–stop planning; TT—train timetabling.

**Model type:** MILP—mixed integer linear programming; MINLP—mixed integer nonlinear programming.

**Headway type:** NA—not applicable.

**Solution method:** ALNS—adaptive large-scale neighborhood search.

### Proposed methods

This study aims to provide the following contributions to the framework of train planning methods.

1. A collaborative optimization approach for stop plans and timetables is proposed in this paper. Two mathematical models are formulated in two phases with consideration of multiple train type. In the first phase, a mixed-integer nonlinear programming model is constructed to obtain a stop plan to minimize the total passenger travel time. In the second phase, the train timetable is optimized based on the stop plan obtained in the first phase. A multiobjective mixed-integer linear optimization model is formulated to minimize the train travel time and the total deviation between the expected and actual departure times from the origin station for all trains.
2. In the process of developing the models, we linked passenger OD demand to train stop planning in the first phase. A decision variable is used to represent the OD passenger flow specific to each train. A train stop planning model is constructed based on the passenger flow assignment. Once the train stop plan is obtained, the train lines need to be rationalized on the time-distance diagram in the second phase. We adopt binary variables to control the train departure sequence from the origin station. A departure time selection matrix based on multiple types of trains is established to constrain the departure sequence of trains. The headway times under different operating conditions are also taken into account in the model.
3. The proposed approach is applied to a real-world case study on a Chinese railway corridor. We use the operating data of the Beijing-Shanghai high-speed railway as model input and adopt the GUROBI optimizer to solve the proposed model. According to the computational results, our models and method generate a more efficient solution for the transport system within an acceptable computation time.

The remainder of this paper is structured as follows. Section 2 presents the problem statements and makes some assumptions. Section 3 gives details of the models in the collaborative optimization approach. In Section 4, the proposed approach is applied to a real case, and the results are analyzed to demonstrate the effectiveness of the models. Some conclusions and future research work are given in Section 5.

## Problem statements and assumptions

As two important parts of a transport plan, a better stop plan and timetable not only meet the passenger demand and provide high-quality transport services but also achieve efficient use of transport resources and reduced operating costs. High-speed railway train stop planning is prepared to determine the stop sequence of trains according to passenger demand, train operation conditions, and station service content. The main task of timetabling is to schedule the departure and arrival times of each train at each station.

**Fig 2:**
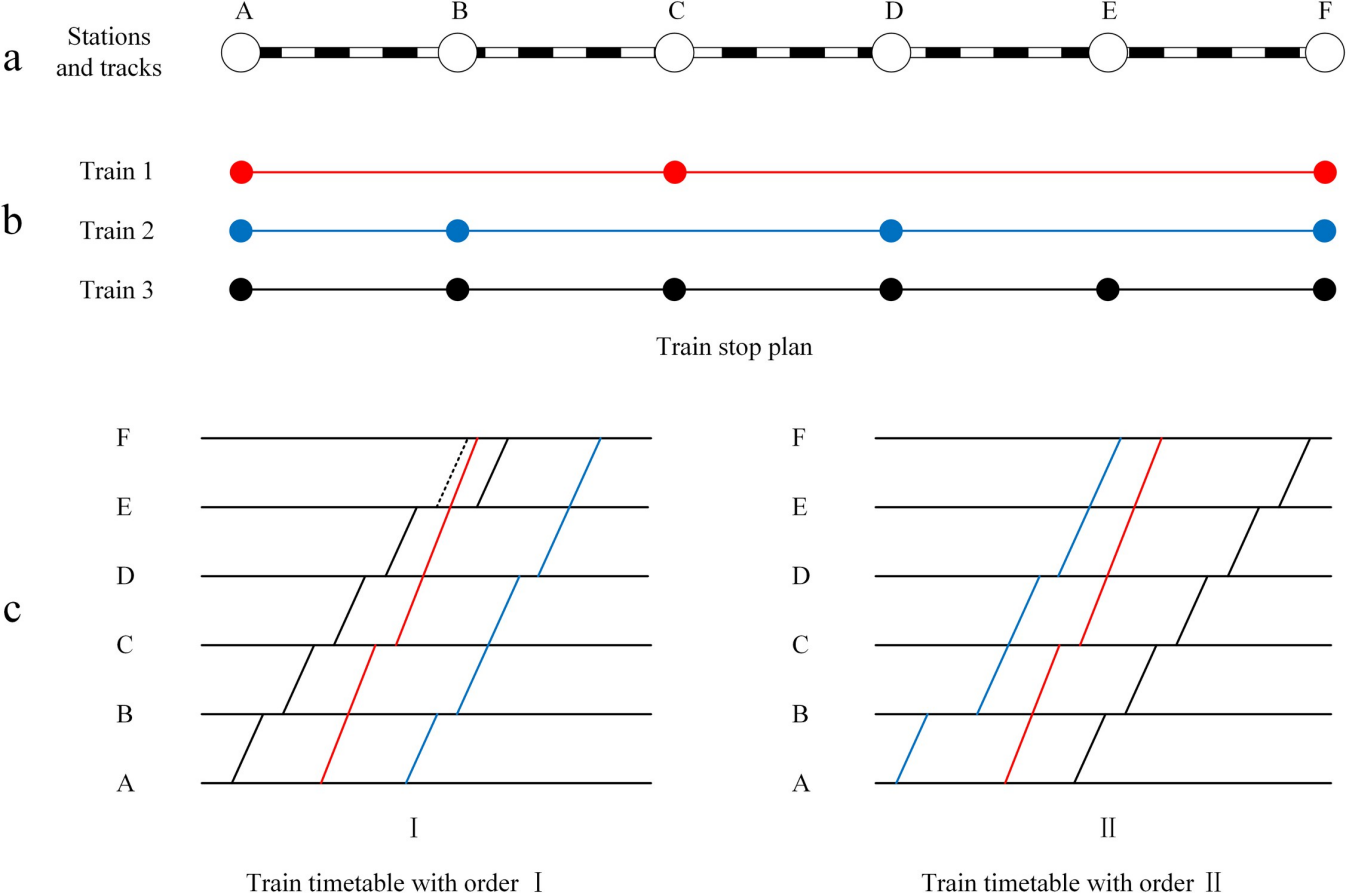
Three levels of the network in high-speed railways.

Therefore, an optimization approach needs to be constructed from three levels of the network, as shown in Fig 2. The first level is the railway infrastructure network, which consists of stations and tracks. The second level is the set of train lines with different stop patterns, which provides travel services for passengers of each OD. Once the stop plan is scheduled, time attributes to each line must be added in the third level. The third level is the train timetable, which consists of the departure and arrival times of each train at each station. A simple network with three trains and six stations is also shown in Fig 2, in which subplots a, b, and c represent the rail infrastructure, stop plan, and timetable, respectively. Three trains operate between the same terminal stations in this railway corridor. The first train with the high-speed class (class *A*) stops only once at station C. The second and third trains are both low-speed class trains (class *B*). The second train stops at stations B and D, while the third train serves all stations.

Clearly, these three trains can provide service for passengers between all ODs. The total travel time of different train services will influence the passenger travel choice with the same OD. For example, all three trains provide transport service from station A to F. Because of the higher speed and fewer stops, the first train is more attractive for passengers from station A to F. When the first train is full, passengers can only choose the last two trains. With the same speed, the travel time of both trains is only affected by the train stop plan. The third train makes two more stops than the second train at stations C and E. More stops increase dwell time, and the corresponding additional acceleration and deceleration time cannot be ignored, especially for high-speed railways. The remaining passengers from station A to F will give priority to the second train with less travel time. Therefore, it is necessary to develop an optimization model that considers passenger assignment and the train stop plan.

Once a train stop plan is generated, the set of train lines needs to be properly aligned on a time-distance diagram. As shown in Fig 2C, there are two timetables obtained from the same stop plan with the different departure orders of the three trains. If the departure sequence from the origin station is train 3—train 1—train 2, train 1 should overtake train 3 at station E to avoid conflict in the section. The dwell time will increase at this point, leading to an extension of the total travel time. There are two strategies to avoid this problem. The first strategy is to adjust the departure sequence of the trains. If the departure sequence from the origin station is train 2—train 1—train 3, there is no overtaking in the timetable, as shown in Fig 2C. The second strategy is to adjust the departure time of trains. Advancing the departure time of train 3 or delaying the departure time of train 1 from the origin station can also avoid overtaking. Therefore, a timetabling model needs to be developed to compute a more efficient and practical solution.

The entire optimization process is divided into two phases. In the first phase, the main objective of a stop planning model is to minimize the total travel time of passengers. From the passenger perspective, the passengers of the same OD may have different train services with different travel times which can affect the selection of train lines during a trip. Travel services with the shortest travel times are more attractive to passengers. The shortest path for passengers will be found in this optimization process. Thus, the problem of passenger assignment based on the shortest path and capacity constraints is embedded into the stop planning model to obtain a system-optimal solution.

In the second phase, the two main objectives for a timetabling model are to minimize the total train travel time and the deviation between the expected and actual departure time from the origin station for all trains. The train travel time is controlled mainly by reducing the dwell time when the running time in the section is fixed. Usually, the timetable of a high-speed railway is relatively fixed over a period of time, which is convenient for passengers to remember and helps to simplify the daily scheduling of the operator, so it is not advisable to change the structure of the timetable to an excessive extent. Therefore, a multitype train departure selection matrix is created to constrain the departure sequence of trains, which controls fewer changes to the structure of the timetable. For example, if a train with a high-speed class departs at 8 am from the origin station, this is also maintained as far as possible in the optimized result. In addition, a timetabling model is proposed to obtain a more efficient result by considering train operation constraints.

To simplify the modeling process, the following assumptions are made.

1. The high-speed railway line studied in this paper is a two-line railway, and the upstream and downstream systems are completely independent. Only one train operation direction is considered in the modeling process.
2. The capacity of the section and station refers to the number of trains that can be arranged per unit time. The capacity constraints can still be satisfied in this study.
3. The study in this paper is based on a single high-speed railway corridor. We assume that all passengers choose trains that can reach their destinations directly.

## Mathematical models

This section will specify each part of the models, including decision variables, parameters, objective functions and systematic constraints. The notations of the parameters and variables are summarized in Table 2.

**Table 2:** Subscript, parameters and variables used in the formulation.

Sets	Definition
*S*	Set of stations in the railway corridor, *s*, *m*, *n* ∈ *S* = {1, 2, …, |*S*|}.
*S*_*i*_	Set of stations on train *i*, *S*_*i*_ = {1, 2, …, |*S*_*i*_|}⊆ *S*.
*T*	Set of trains, *i*, *j* ∈ *T =* {1, 2, …, |*T*|}.
Parameters	Definition
*t*_*ac*_	Additional acceleration time.
*t*_*de*_	Additional deceleration time.
*C*	Train seat capacity.
*μ*	Maximal seat occupancy rate.
*P*_*m*,*n*_	Passenger demand between stations *m* and *n*.
$t_{i,m}^{p}$	Pure running time of train *i* from station *m* to station *m+*1.
*t*_*dwell*_	Minimum dwell time of trains at each station.
*t*_*j*_	Departure time for train *j* from the origin station from original timetable.
$t_{i}^{m,n}$	Travel time of train *i* from station *m* to station *n*.
Δ*t*	Maximum deviation between the expected and actual departure time from the origin stations.
*U*	A large positive number
$\underline{f^{m}}$	Minimum service frequency required for station *m*.
$\bar{f^{m}}$	Maximum service frequency required for station *m*.
$\underline{num_{i}}$	Minimum number of stops for train *i*.
$\bar{num_{i}}$	Maximum number of stops for train *i*.
*h*_*dp*_	Minimum headway between two adjacent trains that the previous train departs from the station and the next train passes through the same station.
*h*_*pd*_	Minimum headway between two adjacent trains that the previous train passes through the station and the next train departs from the same station.
*h*_*pp*_	Minimum headway between two adjacent trains passing through the same station.
*h*_*dd*_	Minimum headway between two adjacent trains departing from the same station.
*h*_*ap*_	Minimum headway between two adjacent trains that the previous train arrives at the station and the next train passes through the same station.
*h*_*pa*_	Minimum headway between two adjacent trains that the previous train passes through the station and the next train arrives at the same station.
*h*_*aa*_	Minimum headway between two adjacent trains arriving at the same station.
*Zmatrix*_*ij*_	Binary parameter limiting the selection of train departure times, which equals 1 indicates that train *i* can select departure time *j*, and 0 otherwise.
Intermediate variables	Definition
*h* ^*dep*^	Minimum headway of two adjacent trains departing from the station.
*h* ^*arr*^	Minimum headway of two adjacent trains arriving at the station.
$t_{i}^{e}$	The expected departure time for train *i*.
Decision variables	Definition
$q_{i}^{m,n}$	Number of passengers taking train *i* from station *m* to station *n*.
$x_{i}^{m}$	Binary variable indicates whether a train stops at a station, which equals 1 if train *i* stops at station *m*, and 0 otherwise.
$a_{i}^{m}$	Arrival time of train *i* at station *m*.
$d_{i}^{m}$	Departure time of train *i* from station *m*.
$y_{ij}^{m}$	Binary variable indicates the train departure order at each station, which equals 1 if train *i* departs from station *m* before train *j*, and 0 otherwise.
*z*_*ij*_	Binary variable indicates the selection of train departure time from the origin station, which equals 1 if train *i* select the time *j* as the expected departure time from the origin station, and 0 otherwise.

### The first phase: Stop planning model

A stop planning model needs to be formulated in the first phase.

Objective function.

The train stop planning model takes the minimum total travel time of passengers as the optimization objective. The total travel time of passengers includes the waiting time at the station and the in-vehicle time. Generally, passengers traveling on high-speed railways buy their tickets in advance and are given departure times. Depending on their travel arrangements, they arrive at the station before the train leaves to make sure they can board the train. Therefore, the waiting time of passengers at the station is not considered, and we only consider the in-vehicle time, which can be formulated as Eq (1). The travel time of the train includes pure running time, dwell time, and additional acceleration and deceleration time, which can also be formulated as Eq (2).


$$Z=\sum_{i=1}^{T}\sum_{m=1}^{|S|-1}\sum_{n=m+1}^{\left|S\right|}q_{i}^{m,n}\cdot t_{i}^{m,n}$$ (1)

$$\begin{array}{l} Z=\sum_{i=1}^{\left|T\right|}\sum_{m=1}^{\left|S_{i}\right|-1}\sum_{n=m+1}^{\left|S_{i}\right|}\sum_{s=m}^{n}t_{i,s}^{p}\cdot q_{i}^{m,n}+\sum_{i=1}^{\left|T\right|}\sum_{m=1}^{\left|S_{i}\right|-1}\sum_{n=m+1}^{\left|S_{i}\right|}\sum_{s=m}^{n}x_{i}^{s}\cdot t_{dwell}\cdot q_{i}^{m,n}\\ +\sum_{i=1}^{\left|T\right|}\sum_{m=1}^{\left|S_{i}\right|-1}\sum_{n=m+1}^{\left|S_{i}\right|}q_{i}^{m,n}\cdot (t_{ac}+t_{bc})+\sum_{i=1}^{\left|T\right|}\sum_{m=1}^{\left|S_{i}\right|-1}\sum_{n=m+1}^{\left|S_{i}\right|}\sum_{s=m}^{n}x_{i}^{s}\cdot (t_{ac}+t_{bc})\cdot q_{i}^{m,n} \end{array}$$ (2)

#### Passenger flow constraints

The passenger flow of each OD must be served. Constraint (3) specifies that the sum of passengers assigned to each train is equal to each passenger OD demand. Constraint (4) indicates that if a train does not provide service between two stations, there is no passenger flow in this train between this OD. U is a large positive number. Constraint (5) specifies that the passenger flow on each train is a nonnegative integer.


$$\sum_{i=1}^{\left|T\right|}q_{i}^{m,n}=P_{m,n},\forall m,n\in S_{i}$$ (3)

$$q_{i}^{m,n}\leq U\cdot x_{i}^{m}\cdot x_{i}^{n},\forall m,n\in S_{i},\forall i\in T$$ (4)

$$q_{i}^{m,n}\in \text{N},\forall m,n\in S_{i},\forall i\in T$$ (5)

#### Seat capacity constraints

Constraint (6) ensures that the passenger flow on each consecutive section of each train is no more than the train seat capacity.


$$\sum_{m=1}^{k}\sum_{n=k+1}^{\left|S_{i}\right|}q_{i}^{m,n}\leq \mu \cdot C,\forall m,n\in S_{i},\forall k\in \{1,2,\ldots ,\left|S_{i}\right|-1\},\forall i\in T$$ (6)

#### Station service frequency constraints

Different classes of stations require different train service frequencies to meet passenger demand. Constraint (7) limits the number of train stops at a specific station to a reasonable range.


$$\underline{f^{m}}\leq \sum_{i=1}^{\left|T\right|}x_{i}^{m}\leq \bar{f^{m}},\forall m\in S$$ (7)

#### Number of train stops constraints

In actual operation, there are different classes of trains on a high-speed railway to meet the travel demand of various passengers. Higher class trains have fewer stops and higher speed, providing faster service for long-distance passengers; while lower class trains have more stops and slower speed, providing service for more passengers of different OD. Passengers can choose the appropriate train service according to their needs. Constraint (8) controls the number of stops of each train within a reasonable range.


$$\underline{num_{i}}\leq \sum_{m=1}^{\left|S\right|}x_{i}^{m}\leq \bar{num_{i}},\forall i\in T$$ (8)

### The second phase: The timetabling model

When the stop plan is determined, a timetabling model needs to be formulated in the second phase.

Objective function. The train timetable model is a multiobjective model. The first objective is to minimize the total train travel time, and the second objective is to minimize the total deviation of the train departure time from the origin station, which can be formulated as Eqs (9) and (10).


$$Z_{1}=\sum_{i=1}^{\left|T\right|}\left(a_{i}^{\left|S_{i}\right|}-d_{i}^{1}\right)$$ (9)

$$Z_{2}=\sum_{i=1}^{\left|T\right|}\left|d_{i}^{1}-t_{i}^{e}\right|$$ (10)

Train departure time selection constraints. It is important to set the departure time of each train from the origin station. A set of expected departure times can be obtained from the original timetable. Constraints (11)–(13) assign a unique departure time to each train from the origin station. There are many types of trains operating on high-speed railways. Some high-speed class trains with few stops usually have a regular departure time, such as every hour. Moreover, high-speed railway train timetables are usually fixed in a period of time to facilitate the memory of passengers and daily operation management. To reduce changes in the structure of an optimized train timetable, some limitations on the departure times of certain trains are needed. Therefore, a select limitation matrix denoted as *Zmatrix*_*ij*_ is proposed to limit the selection of departure times for each train. All trains are divided into several types according to the terminal stations and operating speed, and each type corresponds to a set of expected departure times. The optimized train departure times from the origin station can only be selected from the set of departure times corresponding to the type to which they belong. Constraint (14) limits the selection of departure times for each train.


$$t_{i}^{e}=\sum_{j=1}^{\left|T\right|}z_{ij}t_{j},\forall i\in T$$ (11)

$$\sum_{i=1}^{\left|T\right|}z_{ij}=1,\forall j\in T$$ (12)

$$\sum_{j=1}^{\left|T\right|}z_{ij}=1,\forall i\in T$$ (13)

$$z_{ij}\leq Zmatrix_{ij},\forall i,j\in T$$ (14)

**Fig 3:**
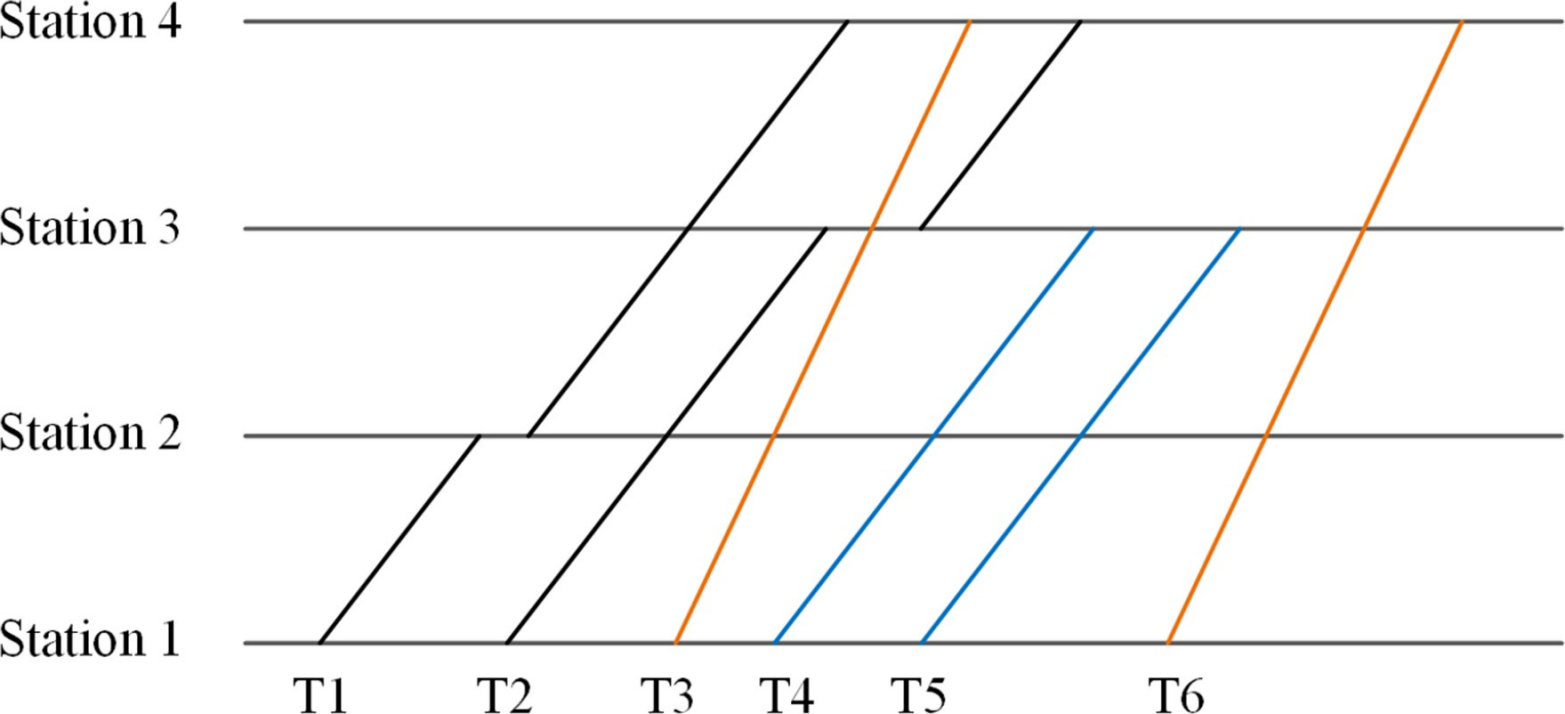
An illustration for timetable with multiple train types.

For example, a timetable with six trains and four stations is shown in Fig 3. The corresponding departure times from the origin station of these trains are *t*_1_, *t*_2_, *t*_3_, *t*_4_, *t*_5_, *t*_6_. All trains can be divided into three types. Class *A* train with terminal stations 1 and 4 is marked as $Type_{A}^{14}=\left\{T_{3},T_{6}\right\}$. Similarly, $Type_{B}^{14}=\left\{T_{1},T_{2}\right\}$ denotes the class *B* train with terminal stations 1 and 4, and $Type_{B}^{13}=\left\{T_{4},T_{5}\right\}$ denotes the class *B* train with terminal stations 1 and 3. The corresponding departure times from the origin station are also divided into three same types. *Zmatrix*_*ij*_ can be formulated as Eq (15). In this matrix, 1 indicates that a train can select this departure time, and 0 means this time cannot be selected by a train.


$$Zmatrix_{ij}\text{=}\left[\begin{array}{cccccc} 1 & 1 & 0 & 0 & 0 & 0\\ 1 & 1 & 0 & 0 & 0 & 0\\ 0 & 0 & 1 & 0 & 0 & 1\\ 0 & 0 & 0 & 1 & 1 & 0\\ 0 & 0 & 0 & 1 & 1 & 0\\ 0 & 0 & 1 & 0 & 0 & 1 \end{array}\right]$$ (15)

#### Interstation travel time constraints

The arrival and departure times of a train at each station can be calculated using Eq (16). The travel time between two stations includes the pure running time and additional acceleration and deceleration time according to the stop.


$$t_{i,m}^{p}+x_{i}^{m}\cdot t_{ac}+x_{i}^{m+1}\cdot t_{bc}=a_{i}^{m+1}-d_{i}^{m},\forall i\in T,\forall m\in \{1,2,\ldots ,\left|S_{i}\right|-1\}$$ (16)

#### Dwelling time constraints

Constraint (17) ensures that the dwell time at a station is greater than a lower bound.


$$d_{i}^{m}-a_{i}^{m}\geq x_{i}^{m}\cdot t_{dwell},\forall m\in S_{i},\forall i\in T$$ (17)

#### Train operation headway constraints

To ensure the safe operation of the railway, it is necessary to formulate train operation headway constraints to maintain a safe distance between adjacent trains. Constraints (18)–(21) ensure a safe headway between adjacent trains at each station. Due to the different operating speeds, overtaking is allowed in the model. A binary variable $y_{ij}^{m}$ indicating the departure sequence of two trains at a station is adopted to avoid train conflicts in sections.


$$d_{i}^{m}+h^{dep}\leq d_{j}^{m}+U(1-y_{ij}^{m}),\forall i,j\in T,i\neq j,\forall m\in S_{i}\cap S_{j}$$ (18)

$$a_{j}^{m+1}+h^{arr}\leq a_{i}^{m+1}+U(1-y_{ij}^{m}),\forall i,j\in T,i\neq j,\forall m\in S_{i}\cap S_{j}$$ (19)

$$y_{ij}^{m}+y_{ji}^{m}=1,\forall i,j\in T,i\neq j,\forall m\in S_{i}\cap S_{j}$$ (20)

$$y_{ijs}\in \{0,1\}$$ (21)

#### Train operation headway calculation

**Fig 4:**
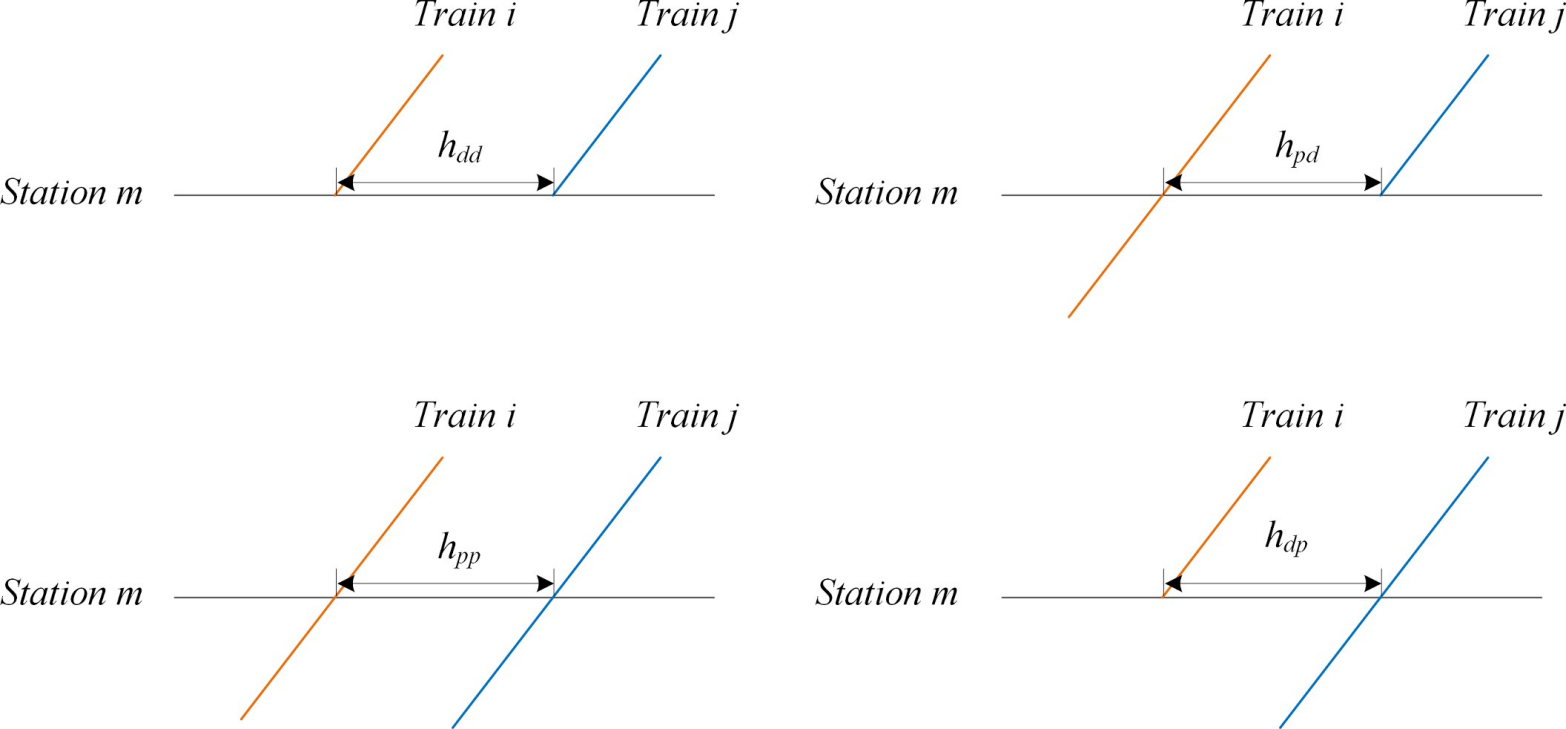
Departure headway in different situation of train stops.

**Fig 5:**
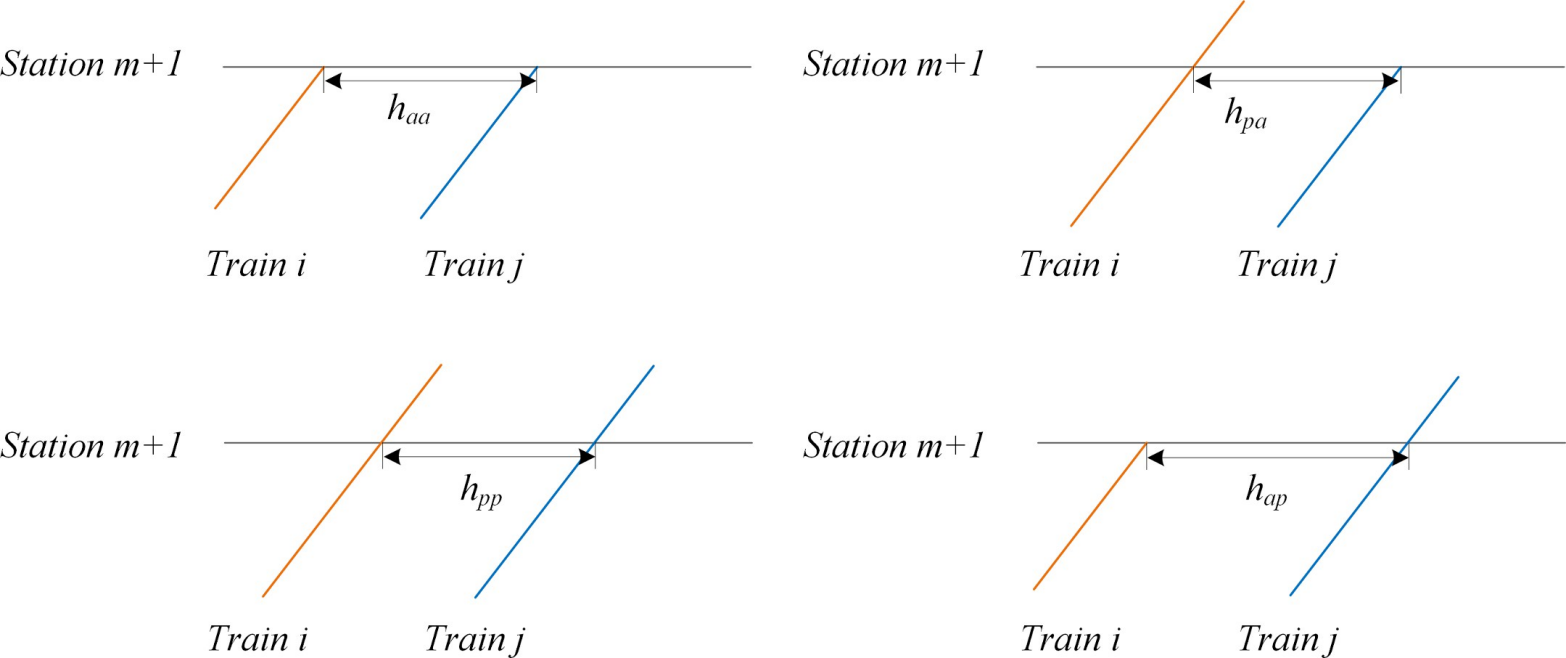
Arrival headway in different situation of train stops.

Train headway is considered more finely in this model, which is different due to the condition of train stops at a station. Figs 4 and 5 show different types of departure headway and arrival headway, respectively. According to the values of $x_{i}^{m}$ and $x_{j}^{m}$, the departure headway can be divided into four types, as shown in Table 3. Similarly, Table 4 represents the four types of arrival headways. The train headway in different conditions can be calculated using Eqs (22) and (23).


$$\begin{array}{l} h^{dep}=x_{i}^{m}\cdot h_{dp}+x_{j}^{m}\cdot h_{pd}+\left(1-x_{i}^{m}-x_{j}^{m}\right)\cdot h_{pp}+x_{i}^{m}\cdot x_{j}^{m}\cdot \left(h_{dd}-h_{dp}-h_{pd}+h_{pp}\right),\\ \forall i,j\in T,i\neq j,\forall m\in S_{i}\cap S_{j} \end{array}$$ (22)

$$\begin{array}{l} h^{arr}=x_{i}^{m}\cdot h_{ap}+x_{j}^{m}\cdot h_{pa}+\left(1-x_{i}^{m}-x_{j}^{m}\right)\cdot h_{pp}+x_{i}^{m}\cdot x_{j}^{m}\cdot \left(h_{aa}-h_{ap}-h_{pa}+h_{pp}\right),\\ \forall i,j\in T,i\neq j,\forall m\in S_{i}\cap S_{j} \end{array}$$ (23)

**Table 3:** Four types of departure headway according to the value of $x_{i}^{m}$ and $x_{j}^{m}$.

$x_{i}^{m}$	$x_{j}^{m}$	Departure headway type
1	0	*h*_*dp*_
0	1	*h*_*pd*_
0	0	*h*_*pp*_
1	1	*h*_*dd*_

**Table 4:** Four types of arrival headway according to the value of $x_{i}^{m}$ and $x_{j}^{m}$.

$x_{i}^{m}$	$x_{j}^{m}$	Arrival headway type
1	0	*h*_*ap*_
0	1	*h*_*pa*_
0	0	*h*_*pp*_
1	1	*h*_*aa*_

#### Departure time from origin station constraints

Constraints (11)-(14) assign an expected departure time to each train. However, it may not be feasible to design a timetable strictly according to the expected departure time. Therefore, we allow the train departure time to fluctuate within a deviation Δ*t*, shown as Constraint (24).


$$t_{i}^{e}-\Delta t\leq d_{i}^{1}\leq t_{i}^{e}+\Delta t,\forall i\in T$$ (24)

## Computational experiments

**Fig 6:**
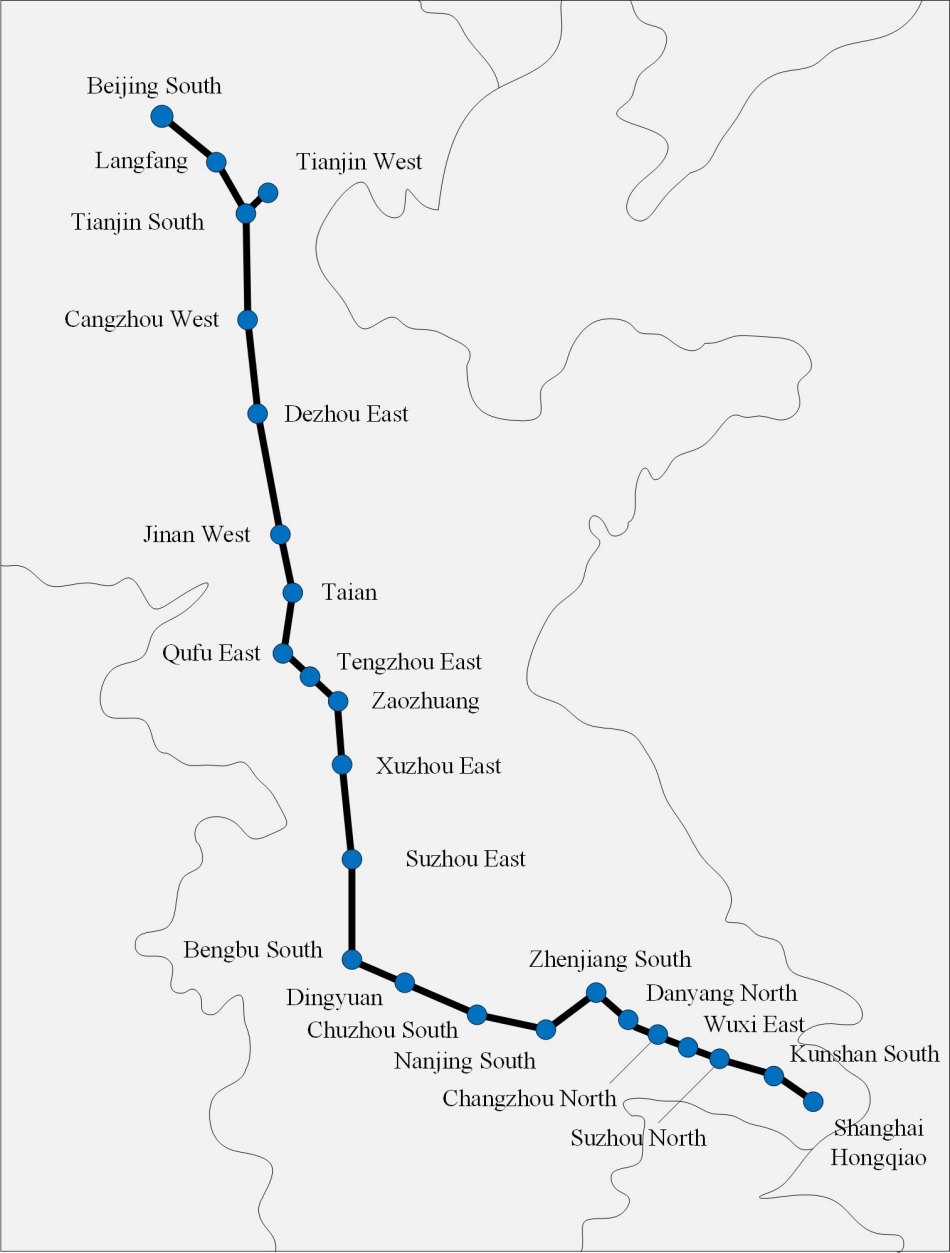
Beijing-shanghai high-speed railway corridor.

In this section, the Beijing–Shanghai high-speed railway in China is considered to verify our approach. Fig 6 shows the Beijing-Shanghai high-speed railway corridor, which is from Beijing South to Shanghai Hongqiao with a total length of 1318 km. This high-speed railway includes 23 stations, of which Beijing South, Tianjin West, Jinan West, Xuzhou East, Nanjing South and Shanghai Hongqiao are the main terminal stations.

### Data preparation and parameter setting

Before optimization, some basic data need to be input, including train operation data, passenger OD demand, and other model parameters.

In this case, we use the train timetable and passenger OD demand from 7:00 to 13:00 on a weekday in 2018 shown in S1 and S2 Tables. The train operation information is shown in Table 5, including the train number, train class, terminal stations and departure times from the origin stations. All trains can be divided into six types according to the terminal stations and operation speed, as shown in Table 6.

**Table 5:** Train operating information from the original timetable.

Train ID	Train class	Departure time	Origin station	Destination station
1	*A*	07:00:00	Beijing South	Shanghai Hongqiao
2	*B*	07:18:00	Jinan West	Shanghai Hongqiao
3	*B*	07:20:00	Beijing South	Shanghai Hongqiao
4	*B*	07:31:00	Xuzhou East	Shanghai Hongqiao
5	*B*	07:50:00	Beijing South	Shanghai Hongqiao
6	*B*	08:05:00	Beijing South	Shanghai Hongqiao
7	*B*	08:15:00	Beijing South	Shanghai Hongqiao
8	*B*	08:35:00	Beijing South	Shanghai Hongqiao
9	*B*	08:50:00	Beijing South	Shanghai Hongqiao
10	*A*	09:00:00	Beijing South	Shanghai Hongqiao
11	*B*	09:07:00	Xuzhou East	Shanghai Hongqiao
12	*B*	09:20:00	Beijing South	Shanghai Hongqiao
13	*B*	09:25:00	Beijing South	Shanghai Hongqiao
14	*B*	09:40:44	Tianjin South	Shanghai Hongqiao
15	*B*	09:50:00	Beijing South	Tianjin South
16	*A*	10:00:00	Beijing South	Shanghai Hongqiao
17	*B*	10:05:00	Beijing South	Shanghai Hongqiao
18	*B*	10:20:00	Beijing South	Shanghai Hongqiao
19	*B*	10:52:05	Tianjin South	Shanghai Hongqiao
20	*B*	11:05:00	Beijing South	Shanghai Hongqiao
21	*B*	11:10:00	Beijing South	Shanghai Hongqiao
22	*B*	11:20:00	Beijing South	Shanghai Hongqiao
23	*B*	11:30:00	Beijing South	Shanghai Hongqiao
24	*A*	12:00:00	Beijing South	Shanghai Hongqiao
25	*B*	12:10:00	Beijing South	Shanghai Hongqiao
26	*B*	12:20:00	Beijing South	Shanghai Hongqiao
27	*B*	12:50:00	Beijing South	Shanghai Hongqiao

**Table 6:** Train types according to train operating speed and terminal stations.

Train type	Train ID
$Type_{B}^{1,23}$	3, 5, 6, 7, 8, 9, 12, 13, 17, 18, 20, 21, 22, 23, 25, 26, 27
$Type_{A}^{1,23}$	1, 10, 16, 14
$Type_{B}^{1,3}$	15
$Type_{B}^{6,23}$	2
$Type_{B}^{3,23}$	14, 19
$Type_{B}^{11,23}$	4, 11

Table 7 shows the pure running time of the two train classes in each section. Due to the speed limit in some railway sections, the calculation of train travel time according to distance and speed is difficult. To simplify the model, the train travel time in a section is obtained from the existing train timetable, which includes pure running time and additional acceleration and deceleration time.

**Table 7:** Pure running time of trains in each section for both speed classes.

Section	Pure running time (min)	
	Class *A* train	Class *B* train
Beijing South-Lang Fang	15	16
Lang Fang-Tianjin South	11	13
Tianjin South-Cangzhou West	16	18
Cangzhou West-Dezhou East	18	20
Dezhou East-Jinan West	17	19
Jinan West-Taian	11	13
Taian-Qufu East	12	14
Qufu East-Tengzhou East	10	12
Tengzhou East-Zaozhuang	6	7
Zaozhuang-Xuzhou East	11	13
Xuzhou East-Suzhou East	12	14
Suzhou East-Benbu South	16	18
Benbu South-Dingyuan	10	11
Dingyuan-Chuzhou	11	13
Chuzhou-Nanjing South	12	13
Nanjing South-Zhenjiang South	13	15
Zhenjiang South-Danyang North	5	6
Danyang North-Changzhou North	6	7
Changzhou North-Wuxi East	10	12
Wuxi East-Suzhou North	5	6
Suzhou North-Kunshan South	6	7
Kunshan South-Shanghai Hongqiao	11	12

Other parameters in the model are shown in Table 8.

**Table 8:** Other parameters in model.

Parameter	Definition	Value
*t*_*ac*_	Additional acceleration time.	2min
*t*_*de*_	Additional deceleration time.	3min
*C*	Train seat capacity.	1000
*μ*	Maximal seat occupancy rate.	100%
*t*_*dwell*_	Minimum dwelling time.	2min
Δ*t*	Maximum deviation between the expected and actual departure time from the origin stations.	10min
*h*_*dp*_	Minimum headway between two adjacent trains that the previous train departs from the station and the next train passes through the same station.	5min
*h*_*pd*_	Minimum headway between two adjacent trains that the previous train passes through the station and the next train departs from the same station.	2min
*h*_*pp*_	Minimum headway between two adjacent trains passing through the same station.	4min
*h*_*dd*_	Minimum headway between two adjacent trains departing from the same station.	5min
*h*_*ap*_	Minimum headway between two adjacent trains that the previous train arrives at the station and the next train passes through the same station.	3min
*h*_*pa*_	Minimum headway between two adjacent trains that the previous train passes through the station and the next train arrives at the same station.	5min
*h*_*aa*_	Minimum headway between two adjacent trains arriving at the same station.	5min

## Results and analysis

In this case, a better service plan is obtained using the proposed collaborative optimization method. We use Python 3.7 and GUROBI (https://www.gurobi.com) version 9.1.2 to solve the models with the given data and parameters. GUROBI is a powerful mathematical programming solver available for optimization problems. GUROBI Optimizer enables users to state their problems as mathematical models and then finds the best solution. For mixed integer programming problems, GUROBI uses the branch and cut algorithm to solve them exactly. The results and analysis of each phase are shown below.

### The first phase

**Fig 7:**
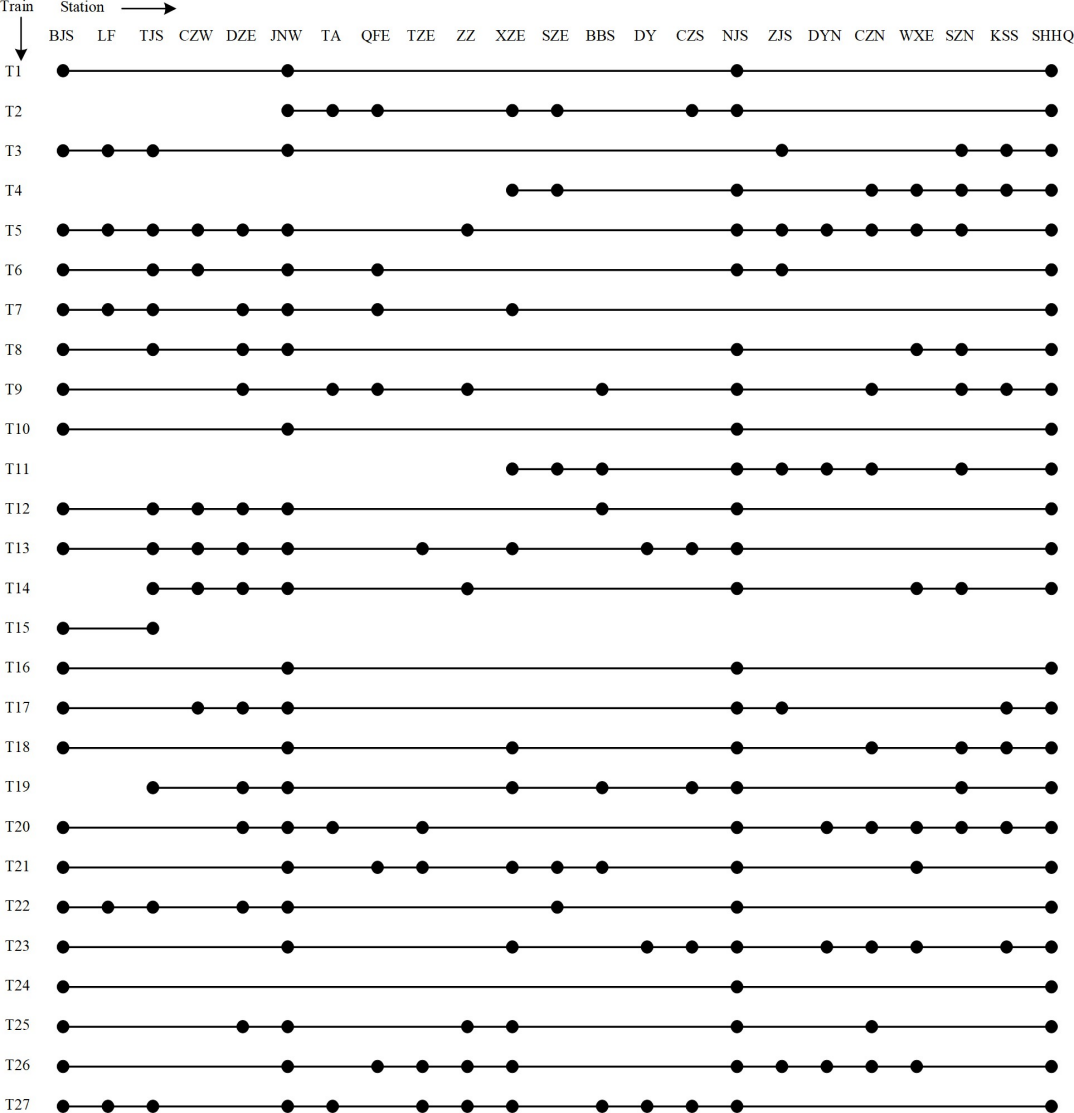
Optimized train stop plan of Beijing–Shanghai high-speed railway.

From the given train information, the passenger OD demand, train seat capacity, and a more efficient stop plan are generated with a gap of 4.13% after 15000 s. Fig 7 shows the specific train stop plan in which a solid dot “•” indicates the stations a train services.

Table 9 reports the total passenger travel time, the number of stops and the average seat occupancy of the original and optimized plans. The average seat occupancy for train *i* can be calculated using Eq (25):

$$\theta_{i}=\frac{\sum_{m=1}^{S_{i}-1}\sum_{n=m+1}^{S_{i}}q_{i}^{m,n}\cdot dis^{m,n}}{C\cdot dis^{i}}$$ (25)

where *dis*^*i*^ and *dis*^*m*,*n*^ represent the total travel distance of train *i* and the distance between stations *m* and *n*, respectively. The total passenger travel time has been optimized from the original 6,694,631 minutes to 6,506,499 minutes, which is a reduction of 2.81%. There are 226 stops in the optimized plan to provide transport services for all passenger ODs, which is 15 fewer than the original plan. Clearly, the optimized average seat occupancy is increased by 4.62%. It can be seen that the optimized stop plan is more efficient while still meeting the passenger demand. Fewer stops will also result in lower operating costs for the operator. Moreover, train seating resources are utilized more efficiently in the optimized solution.

**Table 9:** Comparison of evaluation indexes for original and optimized stop plans.

	Total passenger travel time	Number of stops	Average seat occupancy
optimized stop plan	6506499	226	85.21%
original stop plan	6694631	241	80.59%

Table 10 provides the average seat occupancy of each type of train. Trains of $Type_{A}^{1,23}$ and $Type_{B}^{1,3}$ with the highest average seat occupancy are the most attractive for passengers. For the other four types, the average seat occupancy decreases with the reduction in the number of operating sections.

**Table 10:** The average seat occupancy of each train type.

Train type	Average seat occupancy
$Type_{A}^{1,23}$	100.00%
$Type_{B}^{1,23}$	83.47%
$Type_{B}^{3,23}$	80.75%
$Type_{B}^{6,23}$	79.05%
$Type_{B}^{11,23}$	70.50%
$Type_{B}^{1,3}$	100.00%

**Fig 8:**
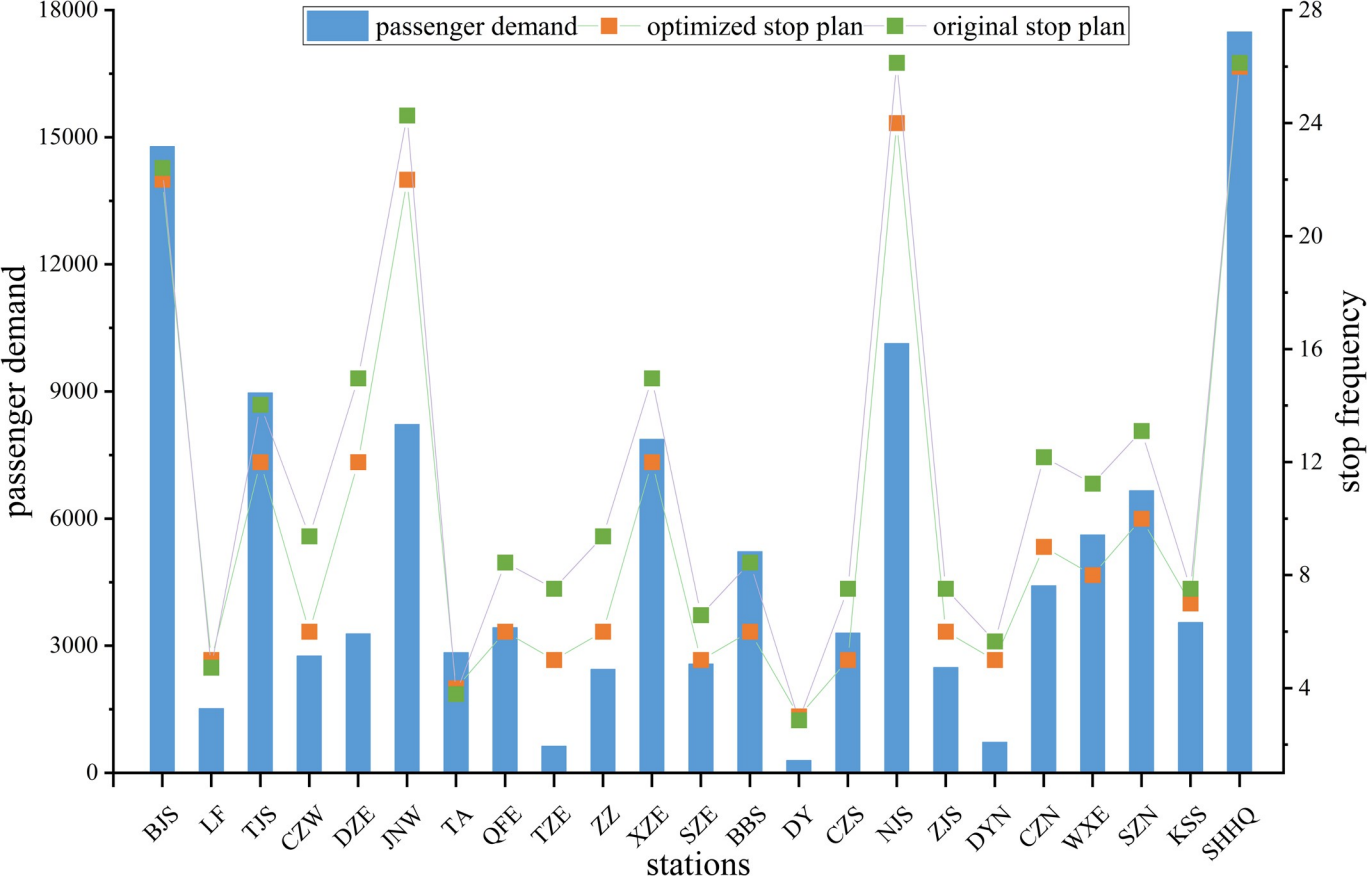
Changes in the frequency of stops at each station and the connection with passenger flow.

Changes in the frequency of stops at each station and the connection with passenger flow is depicted in Fig 8. This demonstrates that the frequency of stops varies with the flow of passengers. The frequency of service has changed at most stations. The frequency of stops at large-sized and medium-sized stations has mostly been reduced since the passenger flow is relatively low and can be met without many stops. Conversely, the frequency of service at some small-sized stations has increased because the low but not concentrated passenger flow needs more services to be satisfied. Therefore, the optimized stopping plan is more in line with the real situation.

### The second phase

**Fig 9:**
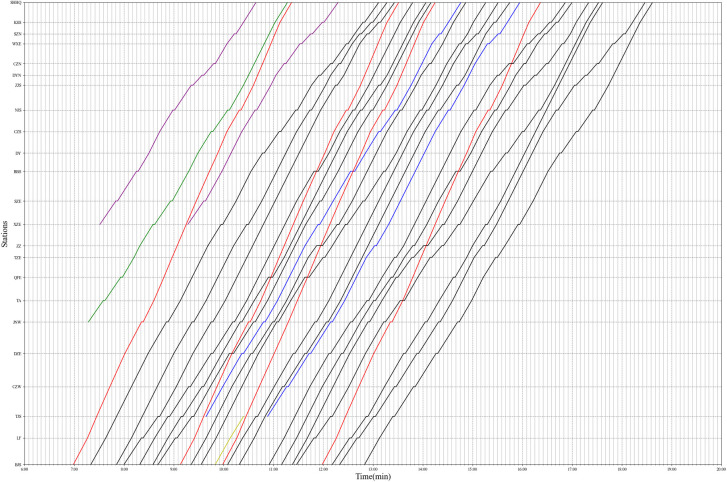
Time-distance diagram of optimized timetable.

Based on the generated stop plan, train operation parameters and expected departure times, a new timetable is obtained from the proposed timetabling model by minimizing the train travel time and departure time deviation. We set *w*_1_ and *w*_2_ as the weights of *Z*_1_ (train travel time) and *Z*_2_ (departure time deviation) respectively. In this experiment, *w*_1_ = 20 and *w*_2_ = 1. The result is obtained after 3600 s and the gap is 0.44%. Fig 9 shows the results of the test, where the different colored lines indicate different types of trains.

Table 11 reports a comparison of the total train travel time, average dwell time and number of overtakings between the original and optimized timetables. The total train travel time has been optimized from the original 8,362 minutes to 8,080 minutes, which is a reduction of 3.34%. The average dwell time is also optimized from 3.09 minutes to 2.19 minutes. The number of overtakings is reduced by 5 times. The results indicate that the proposed model generates a better solution in actual operation.

**Table 11:** Comparison of evaluation indexes for original and optimized timetables.

	Total train travel time (min)	Average dwell time (min)	Number of overtaking
optimized timetable	8080	2.19	10
original timetable	8362	3.09	15

Table 12 gives the actual and expected departure times of each train from the origin station. The total departure time deviation are 69 minutes. The departure times of most trains are adjusted within 10 minutes, in which 9 trains maintained their departure times after optimization.

**Table 12:** The actual and expected departure times of each train from the origin station.

Train ID	Expected departure time	Actual departure time	Time deviation	Train ID	Expected departure time	Actual departure time	Time deviation
1	7:00:00	6:59:00	0:01:00	15	9:50:00	9:50:00	0:00:00
2	7:18:00	7:17:00	0:01:00	16	10:00:00	9:59:00	0:01:00
3	7:20:00	7:20:00	0:00:00	17	10:05:00	10:05:00	0:00:00
4	7:31:00	7:31:00	0:00:00	18	10:20:00	10:19:00	0:01:00
5	7:50:00	7:51:00	0:01:00	19	10:52:05	10:52:05	0:00:00
6	8:05:00	8:00:00	0:05:00	20	11:05:00	10:55:00	0:10:00
7	8:15:00	8:19:00	0:04:00	21	11:10:00	11:10:00	0:00:00
8	8:35:00	8:35:00	0:00:00	22	11:20:00	11:23:00	0:03:00
9	8:50:00	8:41:00	0:09:00	23	11:30:00	11:28:00	0:02:00
10	9:00:00	9:08:00	0:08:00	24	12:00:00	11:59:00	0:01:00
11	9:07:00	9:17:00	0:10:00	25	12:10:00	12:11:00	0:01:00
12	9:20:00	9:21:00	0:01:00	26	12:20:00	12:20:00	0:00:00
13	9:25:00	9:33:00	0:08:00	27	12:50:00	12:50:00	0:00:00
14	9:40:44	9:38:44	0:02:00				

**Fig 10:**
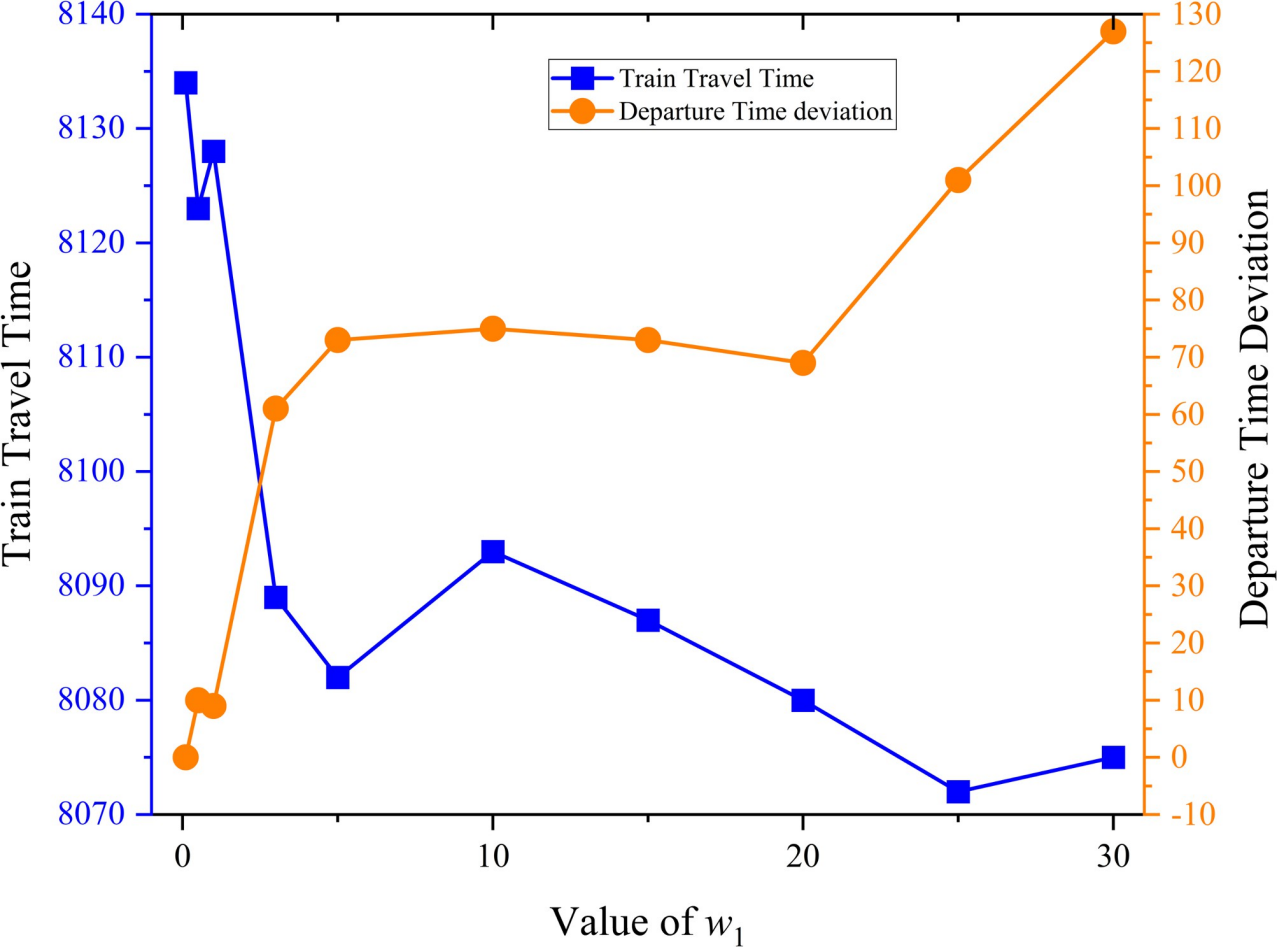
Contrast between train travel time and departure time deviation.

The weight of the total train travel time in the presented model has an effect on the actual departure time in the solution. Finally, we performed some sensitivity analysis to compare the impact of the two objective in timetabling model. A series of experiments using the data in the second phase is proposed, in which *w*_1_ is different and *w*_2_ is fixed to 1. The computation time is set to 3600s. Table 13 shows the experimental results. The contrasts of train travel time and departure time deviation is shown in Fig 10.

**Table 13:** The results of the series of experiments.

*w*_1_	Train travel time	Departure time deviation	Gap
0.1	8134	0	1.07%
0.5	8123	10	1.15%
1	8128	9	1.09%
3	8089	61	0.76%
5	8082	73	0.60%
10	8093	75	0.65%
15	8087	73	0.54%
20	8080	69	0.44%
25	8072	101	0.35%
30	8075	127	0.39%

In Fig 10, with increasing *w*_1_, the departure time deviation becomes larger and train travel time becomes shorter. When *w*_1_ is large, it is necessary to reduce the limit of train departure time to meet the shorter travel time, so the departure time deviation will become larger. Hence, train travel time and departure time deviation can be balanced by adjusting the weight of the objective.

## Conclusions

Aiming at the problem of train stop planning and timetabling, this study proposes a collaborative optimization approach that contains two phases. A stop planning model was constructed by minimizing the total passenger travel time in the first phase. The constraints of OD passenger flow, train seat capacity and number of stops were considered in the stop planning model. In the second phase, a timetabling model was formulated with two objectives, which were to minimize the total train travel time and the total deviation between the expected and actual departure times from the origin station for all trains. We considered a set of train operation constraints to ensure safe train operation, while departure time selection constraints based on multiple types of trains are proposed to control the changes in the optimized timetable. According to a real-world case study, the proposed approach generated a train plan that is more efficient for both passengers and operators.

In future research, the following three areas can be seen as priorities. (1) Unfixed train numbers need to be considered in the model. We need to add or remove trains to accommodate significant changes in passenger flow, such as during holidays. (2) It is meaningful to integrate the two-phase problem into a single model with multiple objectives. Then multiple non-dominated solutions will be obtained for decision-making. (3) The efficient heuristic algorithms can be developed to solve the model, as the efficiency of GUROBI Optimizer is relatively low in solving larger-scale real-world problems.

## Supporting information

S1 Table Original timetable. (XLSX)

S2 Table Passenger OD demand. (XLSX)
